# Achieving optimal technology and behavioral uptake of single and combined interventions of water, sanitation hygiene and nutrition, in an efficacy trial (WASH benefits) in rural Bangladesh

**DOI:** 10.1186/s13063-018-2710-8

**Published:** 2018-07-06

**Authors:** Sarker Masud Parvez, Rashidul Azad, Mahbubur Rahman, Leanne Unicomb, Pavani K. Ram, Abu Mohd Naser, Christine P. Stewart, Kaniz Jannat, Musarrat Jabeen Rahman, Elli Leontsini, Peter J. Winch, Stephen P. Luby

**Affiliations:** 1 Environmental Intervention Unit, Enteric and Respiratory Infections Program, Infectious Diseases Division, International Centre for Diarrhoeal Disease Research, Bangladesh (icddr.b), Dhaka, Bangladesh; 2 School of Public Health and Health Professions, University of Buffalo, Buffalo, NY USA; 3 Rollins School of Public Health, Emory University, Atlanta, GA USA; 4 Department of Nutrition, University of California, Davis, CA USA; 5 Department of International Health, Social and Behavioral Interventions Program, Johns Hopkins Bloomberg School of Public Health, Baltimore, USA; 6 Division of Infectious Diseases and Geographic Medicine, Stanford University, Stanford, CA USA

**Keywords:** WASH benefits, Intervention uptake, Behavior change, Water quality, Sanitation, Handwashing, Child nutrition, Efficacy, Cluster randomized controlled trial, Bangladesh

## Abstract

**Background:**

Uptake matters for evaluating the health impact of water, sanitation and hygiene (WASH) interventions. Many large-scale WASH interventions have been plagued by low uptake. For the WASH Benefits Bangladesh efficacy trial, high uptake was a prerequisite. We assessed the degree of technology and behavioral uptake among participants in the trial, as part of a three-paper series on WASH Benefits Intervention Delivery and Performance.

**Methods:**

This study is a cluster randomized trial comprised of geographically matched clusters among four districts in rural Bangladesh. We randomly allocated 720 clusters of 5551 pregnant women to individual or combined water, sanitation, handwashing, and nutrition interventions, or a control group. Behavioral objectives included; drinking chlorine-treated, safely stored water; use of a hygienic latrine and safe feces disposal at the compound level; handwashing with soap at key times; and age-appropriate nutrition behaviors (pregnancy to 24 months) including a lipid-based nutrition supplement (LNS). Enabling technologies and behavior change were promoted by trained local community health workers through periodic household visits. To monitor technology and behavioral uptake, we conducted surveys and spot checks in 30–35 households per intervention arm per month, over a 20-month period, and structured observations in 324 intervention and 108 control households, approximately 15 months after interventions commenced.

**Results:**

In the sanitation arms, observed adult use of a hygienic latrine was high (94–97% of events) while child sanitation practices were moderate (37–54%). In the handwashing arms, handwashing with soap was more common after toilet use (67–74%) than nonintervention arms (18–40%), and after cleaning a child’s anus (61–72%), but was still low before food handling. In the water intervention arms, more than 65% of mothers and index children were observed drinking chlorine-treated water from a safe container. Reported LNS feeding was > 80% in nutrition arms. There was little difference in uptake between single and combined intervention arms.

**Conclusions:**

Rigorous implementation of interventions deployed at large scale in the context of an efficacy trial achieved high levels of technology and behavioral uptake in individual and combined WASH and nutrition intervention households. Further work should assess how to achieve similar uptake levels under programmatic conditions.

**Trial registration:**

WASH Benefits Bangladesh: ClinicalTrials.gov, identifier: NCT01590095. Registered on April 30, 2012.

## Background

### Uptake of technologies and behaviors in WASH efficacy, effectiveness, and implementation studies

Water, sanitation and hygiene (WASH) interventions have been implemented for many years, and are commonly viewed as an essential part in health and development interventions supported by governments, donor organizations, and development banks. Currently, the specific impact of WASH interventions on disease transmission [1–3], environmental enteric dysfunction, and nutrition and child development outcomes [4–6] is a priority area of research. However it has been difficult to attain sufficient uptake of WASH interventions in effectiveness trials and routine programs implemented at scale [7–9], to replicate the impacts seen in small, well-controlled studies [10, 11].

Efficacy research examines the impact of interventions under ideal or optimal conditions where providers are adequately trained, closely monitored and the respondents are often homogenous. In contrast, effectiveness research examine whether the interventions produce results under near real-world conditions among heterogeneous populations [12]; while implementation research promote the uptake of specific study findings into routine practice [13]. There are questions concerning uptake of WASH technologies (latrines, handwashing stations, water treatments) and WASH behaviors (latrine maintenance and use, handwashing with soap at key times, water treatment and safe storage) for all three kinds of studies. In WASH efficacy studies, researchers ask whether adequate uptake of technologies and behaviors was achieved for the study to qualify as a true efficacy study [14]. Uptake targets are set at a very high level, such as 80% or 90%, and interventions are intensive and carefully monitored [15].

### Effect of WASH combined interventions on uptake

Even under optimal conditions for WASH intervention delivery, there are questions concerning the effects of combined interventions, in comparison to individual interventions, on the quality of work by field workers installing technology and promoting behavior change at the household level, to achieve uptake. Combined interventions are potentially more efficient than individual-component interventions but there is a concern that more intervention messages will dilute effectiveness and thwart uptake levels. Practicing multiple behaviors is more difficult and this may limit the potential for sustained adoption of the behaviors [16, 17]. In addition, and importantly from a behavior change perspective, water, sanitation, handwashing, and nutrition behaviors are quite distinct, with respect to factors such as cues, complexity, timing, and relevant individuals in the household and could require distinct tailor-made promotional strategies.

### Objectives

The WASH Benefits Bangladesh trial examines the impact of WASH and nutrition interventions alone and in combination on outcomes including reported diarrhea and linear child growth [18, 19]. The current analysis aimed to assess the degree of technology and behavioral uptake among participants in the trial, as part of a three-paper series on WASH Benefits Bangladesh Intervention Delivery and Performance.

Implementation quality affects intervention uptake [20]. It is influenced by the standards of the intervention delivery system [21] and the degree of implementation fidelity, which is an important fundamental tool for assessing the implementation process [22–24] and can help to explain the association between intervention and outcomes [25].We describe the WASH Benefits intervention delivery system in the first paper of this series [14] and the system for monitoring implementation fidelity in the second paper in this series [15]. This paper examines whether the study team was able to attain the ambitious targets for uptake of WASH technologies and behaviors required for intervention delivery under ideal conditions, atypical of routine programs.

## Methods

### Study setting and population

The WASH Benefits Bangladesh trial was conducted in four rural districts (Gazipur, Kishorgonj, Mymensingh, Tangail) in central Bangladesh (ClinicalTrials.gov, NCT01590095). These areas were selected based on low iron and arsenic levels in the drinking water (to avoid interference with the chlorine-based water treatment) and absence of major water, sanitation, or focused nutrition programs delivered by the government or nongovernmental organizations (NGOs). Tubewells are the primary source of drinking water and found near most household compounds, and supply groundwater that is generally much less contaminated than surface water, but still commonly contaminated [26]. Handwashing with soap is an existing but erratic practice [27–29]; around 25% of residents washed their hands with soap after defecation and cleaning a child’s anus and less than 1% practiced before preparing food [7]. Open defecation is not extensively practiced by adults [30, 31]; around 11% found ever practiced during baseline. However, only about half the population has access to improved sanitation facilities that hygienically separate human excreta from human contact [32]. Malnutrition in Bangladesh is still high and estimated that approximately 36% of children under 5 are stunted [33]. The subject population was households or compounds with pregnant women and their children who were born within approximately 6 months of the trial’s baseline survey.

The study protocol was approved by the Ethical Review Committee at The International Centre for Diarrhoeal Disease Research, Bangladesh (PR-11063), the Committee for the Protection of Human Subjects at the University of California, Berkeley (2011–09-3652), and the Institutional Review Board at Stanford University (25863).

### WASH benefits intervention design

The WASH Benefits study design and rationale are described in detail elsewhere [18]. In Bangladesh enrolment began in May 2012 during which we identified six to eight closest pregnant women in their second and third trimester to form a cluster. A buffer zone of a minimum of 1 km or 15 min walking distance was enforced before enrolling the next cluster, to minimize spillover between enrolled clusters that might have reduced the risk of disease transmission and reduced the possibility of coping chance among the control clusters from intervention [34]. Eight geographically proximate clusters were grouped together to form a trial block. The trial enrolled 5551 households in 90 blocks (720 clusters). We randomly allocated the clusters of each trial block to one of six intervention arms and retained two clusters as controls. The six interventions were drinking water treatment and safe storage, sanitation, handwashing, and child nutrition, in individual and combined arms, and included free provision of enabling technologies and supplies integrated with parallel behavior change promotion. The water technologies comprised household-level chlorination with sodium dichloroisocyanurate tablets (Aquatabs™ Medentech, Wexford, Ireland) coupled with 10 L safe storage in a covered, narrow-mouth container, based on successful previous trial in rural Bangladesh [26]. The sanitation intervention targeted all households of the index child’s compounds in the sanitation arm, and combined arms (WSH and Nutrition+WSH); these compounds contained a shared courtyard. This compound-based intervention aimed to improve sanitary condition of the shared environment. The sanitation intervention included provision of concrete ring-based double pit pour-flush latrines that had a slab, water seal, and a superstructure for privacy; a potty for young children and a sani-scoop for removal of child feces from the environment. The handwashing intervention households received two handwashing stations, one for the latrine (40 L water reservoir) and one for the kitchen (16 L water reservoir) area; soapy water bottle was provided with a regular supply of detergent sachets to make soapy water. This prototype was tested in our previous study in a similar setting [35, 36]. The nutrition intervention was designed for index children (6–24 months) only with monthly supply of lipid-based nutrient supplement (LNS; Nutriset, France).

Behavioral recommendations were developed based on theory and evidence-based behavioral framework, the Integrated Behavioral Model for Water, Sanitation, and Hygiene (IBM-WASH) model [37]. This framework ensured that we explicitly considered the multiple dimensions (contextual, psychosocial, and technical) and the multiple aggregate levels (societal, communal, interpersonal, individual, and habitual) of determinants of WASH-related behaviors; targeted to the index mother and children. The behavior change messages focused on treatment and safe storage of drinking water for children aged < 36 months, use of latrines for defecation, and the removal of human and animal feces from the compound, handwashing with soap at critical times around food preparation, defecation, and contact with feces, and use of LNS for children aged 6–24 months and age-appropriate nutrition behaviors (pregnancy to 24 months).

The WASH Benefits final intervention design was developed through formative research to account for local context and preferences followed by piloting and repeated iterations. Locally recruited female CHWs who were residents of the study villages received extensive training to deliver and promote the interventions during regular home visits. The intervention approach was not primarily focused on message delivery, but rather emphasized the promoters closely working with the mothers to overcome to targeted behaviors.

The interventions theory of change was based on the importance of an enabling environment created by the various WASH technologies offered and the CHWs’ frequent visits of motivational counseling and problem-solving, to allow behavior change to occur at the household level, provide the stable context needed for habit formation at the sub-individual (habitual) level, change WASH-related social norms at the compound level, and enable nurture toward the children to be expressed at its fullest by the caregiver. These behavioral determinants are summarized in a conceptual framework, IBM-WASH, developed by the same team, based on prior formative research, a multi-level ecological model that specifically identifies the importance of technological determinants out of the general contextual factors in influencing behavior change at all levels of the model. The CHWs received and tried out the technologies and behaviors first, in their own homes. They were thus able to model them and point out their benefits to the mothers, based on personal experience. This draws on key elements for the promotion of self-efficacy: modeling of behaviors, repetition, and practice [38]. The benefits promoted included ease and convenience, attractiveness, recommendation by a well-respected health organization, and health and safety for their baby because of improved hygiene.

CHWs received extensive training at the beginning along with quarterly refreshers to deliver and promote the interventions during regular home visits. Shortly after the training, a community meeting was convened to introduce the intervention and present the promoters and their roles to the community and mothers. Each of the visits was structured with specific objectives and guidelines; the activities included training hardware maintenance, discussion, storytelling, songs in different visits. They engaged in dialogue with the caregivers, rather than conducting on-way message delivery. They observed the WASH situation in the home and compound, listened to the problems faced by the mother, and offered their best advice. Behavioral recommendations were explained by CHWs during home visits, when they also verified that people followed behavioral recommendations, and intervened/problem solved when they did not follow the recommendations.

Each CHW was responsible for one cluster of one intervention arm; there were no promoters in the control communities. The CHWs were instructed to visit the intervention households at least once weekly in the first 6 months and then once in every 2 weeks throughout the study period. They have visited more than that and did have a large presence in the community; which may affect the uptake. However, CHWs did not collect any outcome measures; this was a separate team and the outcome measures were objective and assessed on unannounced follow-up visits, which is why the collected outcome measures were not prone to courtesy bias. These CHWs received a stipend equivalent to USD 20 per month provided by the project, a compensation that is similar to the 5 days of local agricultural labor.

In order to accumulate sufficient intervention-specific promoters to support an efficient training session, up to 3 months elapsed between the baseline assessment and the initiation of intervention hardware distribution. For single intervention, the hardware and household visits were conducted shortly after training. For combined intervention, the sanitation intervention was introduced first followed by handwashing, water treatment, and nutrition. The intervention was implemented along a timeline so that the index child born in an intervention household would be born into a household with the intervention in place.

Problem-solving related to behavioral adoption, and the high CHW to household ratio (1:8) was a defining feature of the theory of change. Since the WASH Benefits Bangladesh was an efficacy study, CHWs did not simply deliver the intervention components and leave it up to the householder to judge whether to adopt the behavioral recommendations or not. The high CHW to household ratio allowed the CHWs to return at frequent interventions, and identify and address the barriers to behavior change through ongoing dialogue with the caregivers. This obviously would not be feasible under routine programmatic conditions. However the trial aimed to assess the impact of adoption of the behavioral recommendations under optimal conditions.

The WASH Benefits efficacy trial intended to achieve optimal intervention uptake including uptake of both the technology and adoption of targeted behaviors. Technology uptake implies “sustained adoption and usage of hardware that was distributed by CHWs”. We defined behavioral uptake as the sustained practice of key behaviors promoted by CHWs.

### Data collection

We assessed uptake from two data sources – monthly fidelity implementation assessments and structured observations; both assessments were unannounced prior to the individual household visits. We designed the data collection tools based on a priori fidelity indicators developed through substantial feedback, comments, and discussions with national and international experts. Monthly fidelity assessment includes spot checks and survey that captured reported behaviors of interest, as well as the presence, functionality, condition, and signs of use of the delivered hardware. Structured observations consisted of the spot checks of technologies plus direct observation of the behavioral practices of interest. No intervention was conducted in control clusters, therefore, those households were not part of the fidelity assessments but were included in the structured observations.

#### Implementation fidelity assessment

Implementation fidelity is defined as the degree to which an intervention is delivered as intended. Health interventions often fail to have an impact because they are not delivered with fidelity [39, 40]. For the current efficacy trial, intervention fidelity was important to ensure that the outcomes could be attributable to the respective intervention. Therefore, the field team conducted unannounced monthly fidelity assessments in a random subset of intervention households. These fidelity assessment included spot checks and surveys over 20 months, from November 2012, 2 months after commencement of intervention delivery, until October 2014. Fidelity assessments were coordinated based on timing of intervention delivery. We surveyed a subset of households based on random selection on a monthly basis.

#### Structured observation

The field workers conducted 5 h of structured observation visits after approximately 15 months of intervention delivery, from February to July 2014. Six (6) trial blocks were randomly selected from each of nine (9) successive implementation phases. From each selected trial block, one (1) household was randomly selected per cluster totaling 324 households from intervention arms (6 blocks*9 phases*6 households) and 108 (6*9*2) households from double-sized control arms. Each observation was approximately 5 h during one of three different time slots (6 am–11 am, 9 am–2 pm and 12 pm–5 pm) to capture activities performed throughout a whole day. The observation times were culturally acceptable for visitors and realistic to observe daily behaviors.

### Analysis

Technology uptake was measured as spot-check indicators (e.g., observed hygienic latrine, presence of residual chlorine in study-provided container). Behavioral uptake, behavior change and use of delivered technology, was reported for some indicators (e.g., LNS feeding) or directly observed for others (e.g. handwashing at key times during structured observation). Contextual spot-check indicators served as proxies for technology and behavioral uptake (e.g., feces presence in the courtyard as an indicator of sani-scoop use and safe feces disposal).

We report intervention uptake and compare rates to the index child’s age to track the proportion of time that the index child in the household was receiving the intervention. To analyze survey and spot check data, we calculated proportions for each indicator and compared uptake in households who received individual versus households who received combined interventions. We compared proportions using the chi-square test adjusted for clustering [41]; the unit of clustering was the geographical cluster. We compared the number of monthly CHW visits in individual versus combined intervention arms using a cluster adjusted t-test, with the same unit of clustering.

To analyze structured observation data, we calculated proportions of each observed behavior across intervention arms. To measure the difference between each intervention and the control arm or between individual and combined interventions, risk difference (RD), 95% confidence interval (CI) and *p* value were calculated using generalized linear models (GLM). We used a clustered sandwich estimator for cluster adjustment; the unit of clustering for this analysis was the repeated events in each observed household.

## Results

**Fig. 1:**
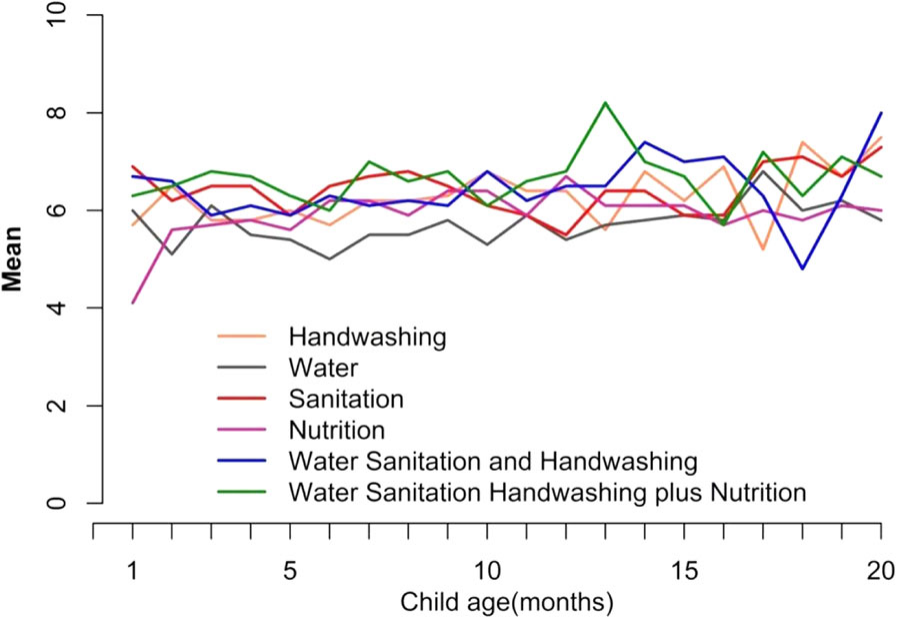
Reported mean number of CHW visits per month by WASH Benefits study arm

There was little difference in social and demographic characteristics between the intervention and control households at baseline (Table 1). The reported number of CHW visits per household per month was high, 5–7 per month (Fig. 1). Each CHW was instructed to visit the assigned intervention households at least once weekly for the first 6 months, then once every 2 weeks.

**Table 1:** Baseline characteristics of WASH Benefits participants in control and intervention arms, rural Bangladesh, 2012

Characteristics	n (%) or mean ± SD						
	Control	Water	Sanitation	Handwashing	Nutrition	WSH^a^	Nutrition+WSH^b^
Education of mother of the youngest child	*N* = 1382	*N* = 698	*N* = 696	*N* = 688	*N* = 698	*N* = 703	*N* = 686
No education	206 (15)	115 (17)	115 (17)	101 (15)	116 (17)	100 (14)	116 (17)
Up-to primary	440 (32)	206 (30)	218 (31)	221 (32)	209 (30)	223 (32)	225 (33)
Above primary	736 (53)	377 (54)	363 (52)	366 (53)	373 (53)	380 (54)	345 (50)
Education of father of the youngest child	*N* = 1378	*N* = 697	*N* = 695	*N* = 687	*N* = 697	*N* = 700	*N* = 685
No education	406 (30)	201 (29)	209 (30)	221 (32)	211 (30)	221 (32)	203 (30)
Up to primary	412 (30)	220 (32)	204 (29)	211 (31)	211 (30)	198 (28)	228 (33)
Above primary	560 (41)	276 (40)	282 (41)	255 (37)	275 (40)	281 (40)	254 (37)
Monthly household income (USD)	133 ± 2.8	140 ± 4.2	131 ± 3.8	127 ± 3.6	132 ± 3.7	140 ± 4.2	137 ± 4.1
People/household	4.7 ± .06	4.7 ± .08	4.7 ± .08	4.7 ± .08	4.7 ± .08	4.7 ± .08	4.7 ± .08
Children <3 years/household	0.2 ± .01	0.2 ± .02	0.2 ± .02	0.2 ± .02	0.2 ± .02	0.2 ± .02	0.2 ± .02
Children <3 years/compound	0.7 ± .02	0.6 ± .03	0.6 ± .03	0.7 ± .03	0.7 ± .03	0.7 ± .03	0.7 ± .03
Own home	1357 (98)	688 (98)	691 (99)	676 (98)	686 (98)	686 (98)	670 (98)
Hectares of owned homestead land (mean ± SD)	.059 ± .002	.058 ± .003	.057 ± .003	.057 ± .003	.063 ± .004	.061 ± .003	.052 ± .002
Hectares of owned agricultural land (mean ± SD)	.427 ± .025	.395 ± .046	.407 ± .029	.411 ± .030	.425 ± .031	.420 ± .028	.459 ± .054
Household have own: n (%)	*N* = 1382	*N* = 698	*N* = 696	*N* = 688	*N* = 698	*N* = 703	*N* = 686
Electricity	784 (57)	422 (61)	408 (59)	405 (59)	409 (59)	426 (61)	412 (60)
Refrigerator	116 (8.4)	52 (7.5)	57 (8.2)	50 (7.3)	56 (8.0)	54 (7.7)	52 (7.6)
Mobile phone	1188 (86)	605 (87)	591 (85)	582 (85)	589 (84)	600 (85)	593 (86)
Television	416 (30)	215 (31)	225 (32)	210 (31)	205 (29)	187 (27)	203 (30)
Motor cycle	100 (7.2)	46 (6.6)	47 (6.8)	35 (5.1)	49 (7.0)	53 (7.5)	32 (4.7)
Observed presence of water and soap in primary handwashing station	289/1256 (23)	149/630 (24)	155/631 (25)	133/622 (21)	149/644 (23)	151/646 (23)	146/640 (23)
Observed presence of water and soap in secondary handwashing station	33/147 (23)	11/78 (14)	15/75 (20)	10/75(13)	11/48 (23)	12/68 (18)	10/72(14)
Reported always handwashing with soap	*N* = 1382	*N* = 698	*N* = 696	*N* = 688	*N* = 698	*N* = 703	*N* = 686
After defecation	590 (43)	288 (41)	298 (43)	271 (39)	289 (41)	334 (48)	287 (42)
After cleaning child’s anus	39 (2.8)	14 (2.0)	24 (3.5)	21 (3.1)	18 (2.6)	28 (4.0)	19 (2.8)
Before food preparation	17 (1.2)	9 (1.3)	6 (0.9)	8 (1.2)	7 (1.0)	11 (1.6)	10 (1.5)
Household owned a toilet	1321 (96)	680 (97)	664 (95)	656 (95)	661 (95)	670 (95)	662 (97)
Observed presence of functional water seal	358 (26)	183 (26)	177 (25)	162 (24)	183 (26)	152 (22)	155 (23)
Observed presence of hygienic latrine	243 (18)	140 (20)	127 (18)	123 (18)	134 (19)	101 (14)	106 (15)
Reported always use of toilet by male	1146 (83)	596 (85)	580 (83)	556 (81)	583 (84)	576 (82)	580 (85)
Reported always use of toilet by female	1283 (93)	665 (95)	649 (93)	625 (91)	648 (93)	651 (93)	647 (94)
Reported last child defecation (<3 years) in potty or toilet	32/272 (12)	16/128 (13)	9/132(6.8)	17/141 (12)	15/131 (11)	16/145 (11)	6/137 (4.4)
Reported safe disposal of <3 years child’s last open defecated feces	14/193 (7.3)	4/92 (4.5)	7/96 (7.3)	5/100 (5.0)	5/90 (5.6)	7/106 (6.6)	8/102 (7.8)
Primary source of drinking water	*N* = 1382	*N* = 698	*N* = 696	*N* = 688	*N* = 698	*N* = 703	*N* = 686
Tubewell	1336 (97)	666 (95)	674 (97)	662 (96)	676 (97)	688 (98)	664 (97)
Piped water	42 (3.0)	31 (4.4)	21 (3.0)	24 (3.5)	20 (2.9)	13 (1.9)	21 (3.1)
Borewell, river, pond, etc.	4 (0.3)	1 (0.1)	1 (0.1)	2 (0.3)	2 (0.3)	2 (0.3)	1 (0.2)
Walking minutes to primary water source (mean ± SD)	.71 ± .04	.71 ± .06	.77 ± .08	.76 ± .07	.70 ± .07	.77 ± .05	.70 ± .07
Reported storage of drinking water	441 (30)	235 (35)	206 (30)	223 (32)	190 (27)	198 (28)	206 (30)
Reported ever treatment of drinking water	10 (0.7)	2 (0.3)	1 (0.1)	2 (0.3)	2 (0.3)	0 (0)	4 (0.6)

^a^WSH: in combination of water quality, sanitation and handwashing interventions

^b^Nutrition+WSH: in combination of water quality, sanitation, handwashing and nutrition intervention

### Sanitation uptake

**Fig. 2:**
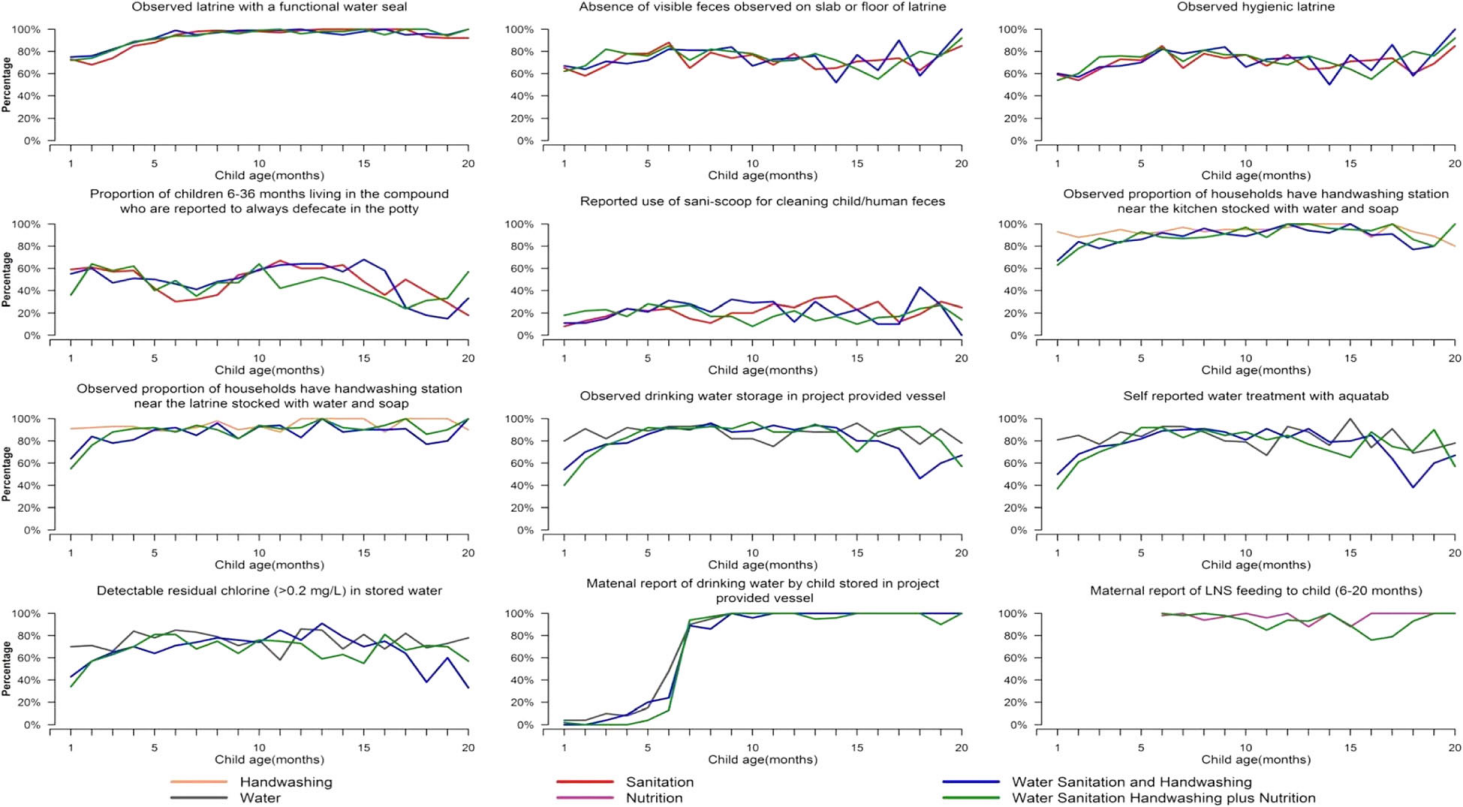
Monthly technology and behavioral uptake measurements over 20 months of intervention delivery, rural Bangladesh, 2012–2014

Among compounds that received the sanitation interventions fieldworkers observed high uptake for presence of hygienic latrine (functional water seal, stool visible on slab/floor), during monthly spot checks (Fig. 2); however, the uptake was slightly lower in the first couple of months. Information on the rapid response triggered by low uptake and resulting improvements in implementation fidelity are reported elsewhere [15]. Uptake was higher among intervention compared to control households for the same indicators (water seal: intervention households: 95–98%, control: 23%, *p* < 0.001; stool visible on slab/floor: intervention households 24–38%, control: 62%, *p* < 0.01; hygienic latrine: intervention households: 60–72%, control: 14%, *p* < 0.001) detected in spot checks during structured observations (Table 2). During structured observation, adults from the sanitation intervention arms more commonly used a hygienic latrine (94–97% of events, *p* < 0.001) compared to adults in the other intervention arms (Table 3). The field workers found moderate use of child potty during child defecation and low use of sani-scoop for cleaning human and animal feces (Fig. 2 and Table 3). Observed safe disposal of human feces was moderate (30–38% of events, *p* > 0.05) (Table 3). Human feces were less commonly observed at the compound (13–26%) among the sanitation intervention households than households in other arms (*p* = 0.01 to 0.57). However, the fieldworkers observed animal feces in the majority of compounds (range 85–96%, *p* > 0.05) across all arms (Table 2).

**Table 2:** Spot checks of sanitation and handwashing facilities during structured observations, rural Bangladesh, 2014

Indicators (%) n	Control	Water		Sanitation		Handwashing		Nutrition		WSH		Nutrition+WSH	
	(%) *N* = 108	(%) *N* = 54	RD^a^ (95%CI) *p* value	(%) *N* = 55	RD (95% CI) *p* value	(%) *N* = 53	RD (95% CI) *p* value	(%) *N* = 54	RD (95% CI) *p* value	(%) *N* = 53	RD (95% CI) *p* value	(%) *N* = 55	RD (95% CI) *p* value
Sanitation observation													
Presence of functional water seal in latrine	(23) 25	(30) 16	6 (−8, 21) 0.38	(95) 52	71 (61, 81) < .001	(32) 17	9 (−6,24), 0.24	(28) 15	5 (−9,19) 0.52	(98) 52	75 (66,84) < .001	(95) 52	71 (61,81) < .001
Stool visible on slab or floor or outside	(62) 67	(69) 37	6 (−9,-22) 0.41	(38) 21	−24 (−39,-8) 0.003	(55) 29	−7 (− 23,9) 0.38	(56) 30	−6 (− 22,-9) 0.43	(26) 14	−35 (−50,-20) < .001	(24) 13	−38 (−52,-24) < .001
Presence of hygienic latrine^b^	(14) 15	(17) 9	3 (− 9,14) .64	(60) 33	46 (30,60) < .001	(19) 10	5 (− 7,17) .43	(19) 10	4 (− 7,17) .46	(72) 38	58 (44,71) < .001	(71) 39	57 (43,70) < 0.001
Human feces observed in the surrounding compound	(30) 32	(19) 10	−11 (−24,2) 0.10	(21) 12	− 8 (− 21,6) 0.27	(32) 17	2 (−12,18) 0.75	(39) 21	9 (− 6,25) 0.24	(13) 7	− 16 (−29,−4) 0.01	(26) 14	-4 (− 18,10) 0.57
Animal feces observed in the compound	(92) 99	(93) 50	1.0 (−8,10) 0.84	(85) 47	−6.2 (−17,4) 0.25	(96) 51	4.6 (− 3,12) 0.22	(94) 51	2.8 (− 5,11) 0.50	(85) 45	−6.8 (− 18,4) 0.23	(89) 49	− 2.6 (− 12,7) 0.61
Handwashing observation													
Handwashing station near the kitchen													
Presence of handwashing station	(83) 90	(85) 46	2 (−10,13) 0.76	(84) 46	.3 (− 11,12) 0.96	(89) 47	5 (−5,16) 0.34	(87) 47	4 (−7,15) 0.50	(89) 47	5 (−5,-16) 0.35	(87) 48	4 (− 7,15) 0.50
Presence of water	(82) 89	(85) 46	3 (−9,14) 0.64	(84) 46	1 (−10,13) 0.84	(75) 40	−12 (−27,1) 0.08	(87) 47	4 (− 7,16) 0.80	(81) 43	−1 (− 14,11) 0.84	(75) 41	−8 (− 21,05) 0.25
Presence of soap/soapy water	(19) 21	(15) 8	−4 (− 16,7) 0.45	(18) 10	−1 (− 14,11) 0.84	(74) 39	52 (38,66) < .001	(26) 14	7 (− 7,2) 0.36	(81) 43	61 (49,74) < .001	(76) 42	57 (43,70) < .001
Presence of water and soap	(19) 21	(15) 8	− 4 (− 16,7) 0.45	(18) 10	− 1 (− 14,11) 0.84	(64) 34	43 (23,58) < .001	(26) 14	6 (− 7,20) 0.36	(76) 40	56 (42,70) < .001	(69) 38	49 (35,64) < 0.001
Handwashing station near the latrine													
Presence of handwashing station	(19) 20	(13) 7	−5 (−17,6) 0.35	(5.5) 3	− 13 (− 22,-3) 0.007	(89) 47	71 (59,81) < .001	(7.4) 4	− 11 (− 21,-1) 0.03	(93) 49	74 (63,84) < .001	(91) 50	72 (62,83) < 0.001
Presence of water	(17) 18	(13) 7	−4 (− 15,7) 0.52	(5.5) 3	− 11 (− 20,-2) 0.01	(74) 39	57 (43,70) < .001	(7.4) 4	−9 (− 19,0) 0.07	(83) 44	66 (54,78) 0.001	(78) 43	61 (48,74) < 0.001
Presence of soap/soapy water	(5.6) 6	(9.3) 5	4 (− 5,12) 0.41	(0) 0	−5 (− 10,-1) 0.01	(79) 42	73 (62,85) < .001	(2.0) 1	−4 (− 9,2) 0.19	(87) 46	81 (71,93) < .001	(82) 45	76 (65,87) < 0.001
Presence of water and soap	(4.6) 5	(9.3) 5	4 (− 4,13) 0.30	(0) 0	− 4 (− 8,-.7) 0.02	(66) 35	61 (48,74) < .001	(2.01) 1	− 3 (− 8,2) 0.31	(77) 41	72 (6,84) < .001	(73) 40	68 (56,80) < 0.001
Presence of water and soap in at least one handwashing station	(21) 23	(22.2) 12	0.9 (−12,14) 0.13	(18) 10	− 3 (− 16,10) 0.63	(77) 41	54 (40,68) < .001	(28) 15	6 (−7,21) 0.37	(85) 45	64 (51,76) < .001	(85) 47	64 (52,76) < .001

^a^RD (risk difference), confidence interval (CI), and *p* value calculated using generalized linear models (GLM) to measure the differences between each intervention arm and the control arms. Clustered sandwich estimator used for cluster adjustment; the unit of clustering was the repeated events in each observed household

^b^Hygienic latrine defined as presence of functional water seal and no visible feces on slab or floor inside

**Table 3:** Structured observation for sanitation, handwashing, water treatment and lipid-based nutrient supplementation (LNS) feeding practices

Indicators n/N (%)	Control	Water		Sanitation		Handwashing		Nutrition		WSH		Nutrition+WSH	
	(%) n/N	(%) n/N	RD^a^ (CI) *p* value	(%) n/N	RD (CI) *p* value	(%) n/N	RD (CI *p* value	(%) n/N	RD (CI) *p* value	(%) n/N	RD (CI) *p* value	(%) n/N	RD (CI) *p* value
Sanitation practices													
Observed adult use of hygienic latrine^b^	(40) 38/94^c^	(44) 16/36	4 (− 31, 39) 0.82	(94) 32/34	54 (28, 79 < .001	(27) 8/30	−14 (− 49,21) 0.44	(31) 13/42	− 9 (− 41,23) 0.56	(97) 37/38	57 (32,82) < .001	(95) 35/37	54 (29,80) < .001
Observed child defecation in potty or hygienic latrine	(32) 22/69	(29) 9/31	−3 (− 49,44) 0.90	(54) 21/39	22 (−18,61) 0.28	(9.1) 2/22	−23 (− 61,-15) 0.24	(5.9) 2/34	− 26 (− 63,11) 0.17	(37) 13/35	5 (− 38,48) 0.81	(40) 16/40	8 (− 31,47) 0.70
Safe disposal of human feces	(16) 12/76	(13) 4/30	−2 (− 32,27) 0.87	(36) 14/39	20 (− 11,51) 0.	(3.2) 1/31	− 13 (− 39,-14) 0.40	(5.3) 2/38	−.11 (− 38,17) 0.45	(38) 13/34	.22 (− 11,56) 0.19	(30) 14/47	.14 (− 16,44) 0.25
Use of sani-scoop for human feces handling	–	–	–	(27) 6/22	–	–	–	–	–	(25) 5/20	–	(38)11/29	–
Use of sani-scoop for animal feces handling	–	–	–	(15) 16/105	–	–	–	–	–	(21) 24/116	–	(12) 12/102	–
Handwashing practices													
Handwashing with soap													
After toilet use	(29) 25/87	(18) 6/34	−11 (−35,13) 0.37	(30) 10/33	2 (−24,27) 0.90	(67) 18/27	38 (11,64) < .005	(40) 16/40	11 (−15,38) 0.41	(74) 26/35	46 (19,72) < .001	(67) 20/30	38 (10,65) < .007
After cleaning child’s anus	(26) 18/69	(39) 12/31	13 (− 7,.32) 0.21	(34) 14/41	.8 (−12,28) 0.43	(61) 14/23	35 (12,58) 0.003	(37) 13/35	11 (− 13,35) 0.36	(69) 24/35	42 (6,78) < .020	(72) 28/39	46 (26,66) < .001
Before infant feeding	(1.8) 6/343	(4.0) 9/227	2 (−2,6) 0.27	(1.9) 3/160	.1 (−2,2) 0.92	(16) 26/161	14 (2,27) 0.02	(2.9) 5/174	1 (−2,4) 0.44	(9.0) 14/155	7 (2,13) 0.008	(5.3) 10/190	4 (−.1,7) 0.06
Before eating	(0.7) 4/546	(.3) 1/296	−.3 (−1,1) .42	(1.5) 4/262	0.7 (−.8,2) .34	(6.9) 21/306	6 (3,9) < .001	(1.7) 5/297	1 (− 1,3) .30	(11) 34/300	11 (5,16) < .001	(5.1) 16/317	4 (1,7) 0.007
Before food preparation	(.5) 1/186	(1.9) 2/104	1 (−2,4) 0.35	(0) 0/106	−.5 (−.1,.5) 0.32	(5.0) 6/121	4 (.4,8.0) 0.03	(0) 0/118	− 5 (−1,.5) 0.31	(8.7) 9/104	8 (2,14) 0.005	(5.0) 6/119	5 (−1,10) 0.11
After cutting vegetables to be cooked	(0.6) 1/161	(3.0) 2/67	2 (−3,8) 0.41	(0) 0/88	−.6 (−1,.6) 0.31	(5.0) 4/80	4 (−.3,9) 0.07	(1.0) 1/100	4 (− 2,3) 0.75	(5.8) 5/86	5 (.1,10) 0.03	(4.2) 4/95	4 (− 1,9) 0.15
After handling raw meat/fish	(6.0) 4/67	(0) 0/23	−6 (−11,-.3) 0.04	(8.6) 3/35	3 (−8,13) 0.63	(44) 8/18	38 (7,70) 0.01	(3.9) 1/26	−2 (− 11,7) 0.65	(18) 3/17	12 (− 9,30) 0.27	(32) 12/38	25 (12,39) < .001
All food handling events	(1.0) 10/960	(1.0) 5/490	−.02 (−1,1) 0.97	(1.4) 7/491	.4 (− 1,2) 0.60	(7.4) 39/525	6 (3,9) < .001	(1.3) 7/541	.3 (− 1,2) 0.72	(10.1)51/507	9 (6,12) < .001	(6.7) 38/569	6 (3,8) < .001
Water practices													
Water treatment-related events													
Proportion of households observed to store water	(51) 55/108	(48) 26/54	−3 (−19,13) 0.74	(49) 27/55	−2 (− 18,14) 0.83	(51) 27/53	.01 (− 16,16) 0.99	(48) 26/54	− 3 (− 19,13) 0.74	(53) 28/53	.02 (− 14,18) 0.82	(62) 34/55	10 (−5,27) 0.18
Storage container fully covered	(18) 13/74	(77) 27/35	60 (41,78) < .001	(38) 16/42	20 (−.3,41) 0.05	(31) 13/42	13 (−9,35) 0.23	(19) 7/37	1 (−16,18) 0.88	(55) 21/38	38 (19,57) < .001	(62) 31/50	44 (24,65) < .001
Water stored with residual chlorination detected^d^	0/70 (0)	(70) 23/33	69 (54,85) < .001	0/41 (0)	0 (− 0,0) 1	0/42 (0)	0 (− 0,0) 1	0/37 (0)	0 (− 0,0) 1	(50) 18/36	50 (33,66) < .001	(65) 30/46	65 (51,80) < .001
Drinking stored water; index child or its mother	(81) 208/258	(97) 167/172	16 (10,23) < .001	(81.3) 113/139	.7 (−8,9) 0.88	(83) 96/116	2 (− 9,12) 0.70	(85)126/148	5 (−5,14) 0.34	(97) 109/113	16 (10,22) < .001	(97) 113/117	16 (10,22) < .001
Drinking stored water; other household members	(87) 222/256	(90) 146/162	3 (−6,12) 0.46	(78.4) 105/134	−8 (−18,2) 0.10	(83) 134/162	−4 (−16,.8) 0.51	(89) 122/137	2 (− 6,10) 0.58	(96) 141/147	9 (3,16) 0.004	(83) 144/173	−3 (−18,12) 0.65
Water collection and storage practices													
Rinsed container with water	(42) 31/74	(60) 21/35	18 (5,42) 0.13	(43) 18/42	1 (−23,25) 0.94	(33) 14/42	−9 (−31,14) 0.46	(41) 15/37	−1 (− 26,26) 0.91	(58) 22/38	16 (−8,40) 0.10	(34) 17/50	− 8 (− 29,14) 0.47
Washed hands with only water	(27) 20/74	(34) 12/35	7 (−14,29) 0.51	(41) 17/42	13 (−11,37) 0.27	(14) 6/42	−13 (− 29,4) 0.13	(24) 9/37	−3 (−23,17) 0.79	(40) 15/38	12 (− 11,36) 0.31	(16) 8/50	− 11 (− 29,7) 0.22
Washed hands with water and soap	(1.4) 1/74	(14) 5/35	13 (.3,26) 0.04	(0) 0/42	−1 (−4,1) 0.31	(0) 0/42	−1 (−4,1) 0.31	(0) 0/37	− 1 (− 4,1) 0.31	(7.9) 3/38	7 (−2,16) 0.16	(0) 0/50	− 1 (− 4,1) 0.31
Serving stored drinking water													
Rinsed glass with drinking water	(15) 76/514	(7.8) 26/334	−7 (−13,-1) 0.02	(20) 55/273	5 (−.4,14) 0.24	(11) 30/278	−4 (−10,2) 0.21	(14) 40/285	−.8 (− 7,6) 0.83	(8.1) 21/260	− 7 (− 13,-1) 0.03	(7.6) 22/290	− 7 (− 13,-2) 0.01
Washed hands with only water	(5.5) 28/514	(4.5) 15/334	− 1 (−5,3) 0.65	(4.4) 12/273	−1 (−6,4) 0.69	(3.2) 9/278	−2 (−6,2) 0.29	(3.2) 9/285	−2 (− 6,2) 0.24	(3.5) 9/260	− 2 (− 6,2) 0.31	(1.4) 4/290	− 4 (− 7,-1) 0.01
Drinking water stored in the study provided container (topaz); index child or its mother	–	(74) 128/172	–	–	–	–	–	–	–	(66) 74/113	–	(68)80/118	–
Drinking water stored in the study provided container (topaz); other household members	–	(40) 65/162	–	–	–	–	–	–	–	(57) 84/147	–	(42) 72/173	–
Nutrition practices													
LNS events													
Observed LNS serving (at least 1)	–	–	–	–	–	–	–	(56) 30/54	–	–	–	(59) 32/54	–
Consumption (index child)													
Consumed 1 full sachet	–	–	–	–	–	–	–	(65) 26/40	–	–	–	(97) 31/32	–
Consumed 2 full sachet	–	–	–	–	–	–	–	(0) 0/40	–	–	–	(0) 0/32	–
Partially from left over sachet	–	–	–	–	–	–	–	(13) 5/40	–	–	–	(0) 0/32	–
Partially eaten and stored	–	–	–	–	–	–	–	(18) 7/40	–	–	–	(0) 0/32	–
Partially eaten and thrown away	–	–	–	–	–	–	–	(5) 2/40	–	–	–	(3.1) 1/32	–
Mother’s hands washed with soap before feeding LNS	–	–	–	–	–	–	–	(13) 5/40	–	–	–	(31) 10/32	–

^a^RD (risk difference), confidence interval (CI), and *p* value calculated using generalized linear models (GLM) to measure the difference between each intervention arm and the control arms. Clustered sandwich estimator used for cluster adjustment; the unit of clustering was the repeated events in individual household

^b^Hygienic latrine defined as presence of functional water seal and no visible feces on slab or floor inside

^c^Denominator was total number of defecation and urination events observed in the HHs which included use of hygienic, non-hygienic latrine and open defecation

^d^Residual chlorine > 0.2 mg/L with the HUCH method

### Handwashing uptake

Among households that received the handwashing intervention, the proportion of households with handwashing stations observed to be stocked with water and soap or soapy water near the kitchen and latrine was high across intervention arms (Fig. 2). Somewhat higher uptake was noted in the first few months of fidelity assessments (Fig. 2). Similarly, during structured observation, field workers observed high uptake in the kitchen (range 64–76%, *p* < 0.001) and latrine area (range 66–77%, *p* < 0.001) (Table 2). Observed handwashing with soap was more common after toilet use (range: 67%–74% of events, *p* < 0.05) and after cleaning a child’s anus (range: 61%–72%, *p* < 0.05) compared to other intervention (range 34–39%) and control households (range 26–29%). However, the field workers observed only 5–11% of participants washing their hands with soap before eating and before food preparation across the handwashing arms (*p* = 0.001 to 0.11) (Table 3).

### Water treatment and safe storage uptake

Observed drinking water stored in study-provided containers and self-reported water treatment with Aquatabs were somewhat lower in the first few months but high later on (Fig. 2). Detectable residual chlorine was lower than self-reported Aquatab treatment but sizeable (Fig. 2). In households that received the water intervention, more than 65% (range 66–74%) of mothers or index children drank treated water from the study-provided containers (Table 3).

### LNS uptake

Among mothers of children aged between 6 to 20 months of age, more than 80% reported LNS feeding (1 or 2 sachets per day) across the nutrition intervention arms (Fig. 2). During structured observation, 56% of index children were observed to consume at least one LNS sachet in the individual nutrition intervention arm and 59% in the combined Nutrition+WSH intervention arm (Table 3).

### Comparison of uptake among individual and combined interventions

Some small differences were detected in the overall uptake between individual and combined interventions in the uptake measurements over 20 months (Table 4).

**Table 4:** Differences in the uptake across individual and combined intervention arms over 20 months

Indicators	Water, % (mean)	Sanitation, % (mean)	Handwashing, % (mean)	Nutrition, % (mean)	WSH, % (mean) *p* value^a^	Nutrition+WSH, % (mean) *p* value^a^
Observed latrine with a functional water seal	–	89	–	–	91 0.54	90 0.80
Absence of visible feces observed on slab or floor of latrine	–	73	–	–	73 0.98	75 0.59
Observed hygienic latrine	–	70	–	–	70 0.97	72 0.63
Proportion of children 6–36 months living in the compound who are reported to always defecate in the potty	–	48	–	–	50 0.69	47 0.75
Reported use of sani-scoop for cleaning child/human feces	–	20	–	–	21 0.71	20 0.90
Mean CHW visits per month in Sanitation arms	–	(6.4)	–	–	(6.3) 0.68	(6.6) 0.35
Observed proportion of households have handwashing station near the kitchen stocked with water and soap	–	–	94	–	86 0.005	87 0.003
Observed proportion of households have handwashing station near the latrine stocked with water and soap	–	–	93	–	85 0.002	87 0.008
Mean CHW visits per month in handwashing arms	–	–	(6.2)	–	(6.3) 0.56	(6.6) 0.052
Observed drinking water storage in project provided container	88	–	–	–	81 0.013	81 0.027
Self-reported water treatment with Aquatab	84	–	–	–	78 0.046	77 0.03
Detectable residual chlorine > 0.2 mg/L in stored water	76	–	–	–	68 0.034	67 0.016
Mother’s report of index child drinking water stored in project provided container	58	–	–	–	54 0.48	51 0.20
Mean CHW visits per month in water arms	(5.6)	–	–	–	(6.3) 0.007	(6.6) 0.000
Self- reported feeding LNS to child (6–20 months)	–	–	–	84	–	84 0.95
Mean CHW visits per month in Nutrition arms	–	–	–	(5.8)	–	(6.6) .002

*CHW* community health worker; *LNS* lipid-based nutrient supplementation; *WSH* water quality, sanitation, handwashing

^a^Cluster adjusted chi-square test for proportion and cluster adjusted *t* test for mean

All sanitation uptake indicators were similar and did not differ significantly for the individual compared to the two combined interventions (*p* = 0.63 to 0.97). In the individual handwashing intervention, the majority (93–94%) of households had water and soap in both handwashing stations (near the kitchen and near the latrine), and this proportion was somewhat higher compared to households that received combined handwashing interventions (range 85–87%, *p* < 0.01). The proportion of self-reported water treatment with Aquatabs (84%) was somewhat higher for those who received the individual water intervention than the combined interventions (range 77–78%, *p* < 0.05). Similarly, detectable chlorine residual (76%) was more common in the individual intervention compared to combined intervention households (range 67–68%, *p* < 0.05). However, mothers’ reports of index children drinking water from the study-provided container were similar across the water intervention arms (range 51–58%, *p* > 0.05) (Table 4).

## Discussion

The assessment of the technology and behavioral uptake in the WASH Benefits efficacy trial demonstrated moderate to high level uptake of desired technologies and behaviors in both individual and combined intervention arms. In some individual arms, we found somewhat higher uptake compared to combined intervention arms for a subset of indicators (fully stocked handwashing stations, water storage in study-provided containers, and self-reported water treatment); however, these uptake differences were small.

### Sanitation uptake

We identified higher proportions of hygienic latrines, absence of visible feces on the latrine slab or floor, presence of functional water seal, and targeted behavioral uptake in the sanitation intervention households compared to households that did not receive any sanitation intervention. However, we found comparatively lower levels of technology and behavioral uptake in child sanitation practices in all sanitation intervention arms. Nonetheless, child sanitation practices in these arms were higher compared to the child sanitation practices achieved in other studies [7, 8, 42, 43] and during the WASH Benefits baseline.

Open defecation among children under the age of five in Bangladesh and elsewhere is common [44–47]. It is possible that the existing open defecation practice among children was so common and accepted that it acted as a barrier to the uptake of potty use, especially since this requires potty training, which can be time-consuming for mothers [48, 49]. Similarly, unsafe child feces disposal is highly prevalent in rural Bangladesh even when a household has latrine access [50]. Unsafe feces disposal decreases if a potty is available in the household, but it is very common for a child under the age of 3 to defecate in a nappy or on the ground [50]. Even when a potty was available and promoted actively in this study, observed use was not very high, and possibly linked to unsafe feces disposal practices. Further research might identify approaches to improve the promotion of child sanitation practices in this and similar settings. Adapting toilets to be child-friendly might improve safe feces management of preadolescent children.

### Handwashing uptake

Earlier studies from Bangladesh reported low uptake of handwashing behaviors at key times [7, 29, 51–54]. Our study identified higher uptake of handwashing stations at designated places, which likely contributed to the improved handwashing practices noted in the structured observation. These findings highlight the importance of a convenient location for acquiring the habit of washing hands [29, 55]. Households were able to maintain the study-provided handwashing station. However, improved but still low behavioral uptake was found before food preparation and infant feeding, consistent with other studies in similar settings [29, 51]. We provided a designated handwashing station near the kitchen, which could have had an impact on the frequency of handwashing [56], though the presence of a handwashing station was not a sufficient condition for achieving behavior change. Further research on how handwashing can be promoted in the absence of intense interpersonal communication and without free provision of supplies is a priority area for handwashing research. The popularity of soapy water suggests it may be a promising component [57].

### Water treatment uptake

The technology and behavioral uptake of the water treatment was similar to another intervention trial in Bangladesh that used the same approach [26]. That study also reported that safe storage alone markedly improved microbiological quality of stored water and subsequently reduced diarrhea. While intervention households commonly treated water with Aquatabs, reported practice was higher than detected free chlorine; higher concentration of iron in water from some tubewells may have reacted with Aquatabs and thereby reducing detectable chlorine [58]. However, supplying Aquatabs at scale would require further research to gauge demand and willingness to pay. Safe water storage alone might be sufficient to improve water quality in some settings [26].

### Nutrition uptake

The high LNS uptake in nutrition interventions was consistent with similar previously implemented interventions [59–63]. Using formative research to develop nutrition intervention messages [64] that were culturally sensitive likely influenced high levels of adoption [65]. This confirms that LNS is highly acceptable in this setting, however nutrition programs will need to determine ways to fund product purchase and distribution.

### Comparison of uptake among individual and combined intervention

Combined interventions showed high technology and behavioral uptake, even though other research has suggested that too many behavior change communication (BCC) interventions risk overwhelming the target audience [16, 17, 66, 67]. Our intensive intervention delivery system, highly trained CHWs, as well as using a phased intervention roll out rather than introducing multiple interventions together in the households [14], may have contributed. Specifically for nutrition and child sanitation practices, where delivery aligned as the children’s cohort aged. The training for children on potty use was age-appropriate. Routine programs, by contrast, have to concurrently serve children of diverse ages and developmental stages, and therefore diverse programmatic needs, requiring the CHWs’ concurrent dissemination of interventions targeted to multiple age groups at once. Future research could explore approaches to deliver complex interventions to larger numbers of children of diverse ages.

CHWs were the cornerstone of intervention delivery where uptake was a primary requirement. The importance of the capacity of health workers to promote complex interventions has been highlighted elsewhere [68]. A common concern has been that increasing health promoters’ workload can result in diluted messages, and receivers of these messages can be overwhelmed [67, 69]. However, in this assessment, we found somewhat lower uptake in combined intervention households, and only for handwashing and water treatment behaviors; this relatively lower uptake level was modest. Mothers in low-income settings setting have time constraints that can limit their time to integrate additional responsibilities into their daily routines [70, 71]; likely to prioritize convenient behavior options. In fact, when it comes to adopting new behaviors, when given a choice, there is evidence that people tend to choose convenience over effectiveness [72]. It is possible that, when multiple behaviors promoted in combination, the amount of attention/effort/time dedicated to these inconvenient and time-consuming behaviors had to be limited and overall uptake of multiple behaviors fell compared to the uptake when these behaviors were promoted individually.

First and foremost, the CHW to population ratio (1:8) was very high; hence the CHWs could demonstrate the enabling technologies and behavioral recommendations in their own homes and the homes of the study participants, thus promoting an in-depth familiarity that likely increased their own self-efficacy to promote the behavioral recommendations. In addition, they received extensive trainings, and close supervision, and conducted repeated household visits that included problem-solving and behavior reinforcement [14].

It is possible that individual and combined interventions were received equally well by the study participants because they were all linked within the common theme of child health and well-being. The multiple messages were all complementary not conflicting/contradictory. Earlier research shows that thinking about a behavioral outcome can occur easily if multiple behaviors are thematically linked [73].

The study has some limitations. Some of the uptake indicators were reported and not directly observed, potentially overestimating uptake. In addition, the presence of an observer might alter practices during the observation period [74] and, therefore, overestimate uptake. We attempted to reduce the limitations of questions on reported behaviors by adding spot checks and residual chlorine measurements. We attempted to reduce the impact of the observer by arriving unannounced and extending the observation into 5 h, which likely minimized the reactivity [75]. In addition, observers had no connection with the intervention to reduce reactivity. In the case of LNS consumption, the 5-hour period may have been too short to observe a feeding event, hence the discrepancy between reported and observed uptake. A limitation of efficacy trials is that the intervention delivered under optimal conditions, and so these findings do not readily generalize to routine programs.

## Conclusions

The WASH Benefits efficacy trial demonstrates that with a carefully designed intervention, explicitly based on a broad behavior change theory and formative research, implemented by well-trained and supervised CHWs, high uptake of water, sanitation, hygiene, and nutrition-related behaviors could be achieved within low-income rural communities. Adapting techniques that were effective in this well-resourced efficacy study, to large-scale programmatic interventions would require a focused research effort and iterative learning, but the high uptakes achieved suggest that such an effort may be worth the investment.

## Abbreviations

BCC: Behavior change communication
CHW: Community health worker
IBM-WASH: Integrated Behavioral Model for Water, Sanitation, and Hygiene
LNS: Lipid-based nutrient supplementation
NGO: Nongovernmental organization
WASH: Water, Sanitation, and Hygiene
